# Preclinical validation of NeoWarm, a low-cost infant warmer and carrier device, to ameliorate induced hypothermia in newborn piglets as models for human neonates

**DOI:** 10.3389/fped.2024.1378008

**Published:** 2024-04-03

**Authors:** Nick D. P. Bluhm, Grant M. Tomlin, Orlando S. Hoilett, Elena A. Lehner, Benjamin D. Walters, Alyson S. Pickering, Kevin Alessandro Bautista, Sherri L. Bucher, Jacqueline C. Linnes

**Affiliations:** ^1^ Weldon School of Biomedical Engineering, Purdue University, West Lafayette, IN, United States; ^2^ Indiana University School of Medicine, Indianapolis, IN, United States; ^3^ Department of Biomedical Engineering, University of Cincinnati, Cincinnati, OH, United States; ^4^ The Elmore Family School of Electrical and Computer Engineering, Purdue University, West Lafayette, IN, United States; ^5^ School of Materials Engineering, Purdue University, West Lafayette, IN, United States; ^6^ Department of Community and Global Health, Richard M. Fairbanks School of Public Health, Indiana University-Indianapolis, Indianapolis, IN, United States; ^7^ Department of Pediatrics, Division of Neonatal-Perinatal Medicine, Indiana University School of Medicine, Indiana University, Indianapolis, IN, United States; ^8^ Department of Public Health, Purdue University, West Lafayette, IN, United States

**Keywords:** low to middle income country (LMIC), neonatal hypothermia, thermal model, piglet model, kangaroo mother care (KMC), vital signs monitoring system (VSMS), neonatal mortality and morbidity

## Abstract

**Introduction:**

Approximately 1.5 million neonatal deaths occur among premature and small (low birthweight or small-for gestational age) neonates annually, with a disproportionate amount of this mortality occurring in low- and middle-income countries (LMICs). Hypothermia, the inability of newborns to regulate their body temperature, is common among prematurely born and small babies, and often underlies high rates of mortality in this population. In high-resource settings, incubators and radiant warmers are the gold standard for hypothermia, but this equipment is often scarce in LMICs. Kangaroo Mother Care/Skin-to-skin care (KMC/STS) is an evidence-based intervention that has been targeted for scale-up among premature and small neonates. However, KMC/STS requires hours of daily contact between a neonate and an able adult caregiver, leaving little time for the caregiver to care for themselves. To address this, we created a novel self-warming biomedical device, NeoWarm, to augment KMC/STS. The present study aimed to validate the safety and efficacy of NeoWarm.

**Methods:**

Sixteen, 0-to-5-day-old piglets were used as an animal model due to similarities in their thermoregulatory capabilities, circulatory systems, and approximate skin composition to human neonates. The piglets were placed in an engineered cooling box to drop their core temperature below 36.5°C, the World Health Organizations definition of hypothermia for human neonates. The piglets were then warmed in NeoWarm (*n* = 6) or placed in the ambient 17.8°C ± 0.6°C lab environment (*n* = 5) as a control to assess the efficacy of NeoWarm in regulating their core body temperature.

**Results:**

All 6 piglets placed in NeoWarm recovered from hypothermia, while none of the 5 piglets in the ambient environment recovered. The piglets warmed in NeoWarm reached a significantly higher core body temperature (39.2°C ± 0.4°C, *n* = 6) than the piglets that were warmed in the ambient environment (37.9°C ± 0.4°C, *n* = 5) (*p* < 0.001). No piglet in the NeoWarm group suffered signs of burns or skin abrasions.

**Discussion:**

Our results in this pilot study indicate that NeoWarm can safely and effectively warm hypothermic piglets to a normal core body temperature and, with additional validation, shows promise for potential use among human premature and small neonates.

## Introduction

There are nearly 15 million premature (babies born <37 weeks gestation) and low-birthweight (less than 2.5 kg) babies born each year (1, 2). 1.5 million of these neonates will not survive, with the vast majority of these deaths occurring in low and middle income countries (LMICs), a staggering—and unacceptable trend—that has persisted for decades (3–8). One of the main contributing factors which underlies high rates of preventable neonatal mortality is hypothermia (9–12). For a variety of reasons related to underdeveloped physiology, as well as environmental conditions both within health facilities and in the home and community settings, premature, low birthweight, and small-for-gestational age newborns struggle to maintain a normal body temperature, defined as 36.5°C–37.5°C (13–15). Newborns have a greater surface area-to-weight ratio, larger head-to-body ratio, and less adipose tissue for insulation, predisposing them to heat loss without appropriate thermal care interventions (16).

In high-resource settings, hypothermia is most often prevented, or treated, through the use of incubators and radiant warmers (17). However, in low-resource settings, incubators and radiant warmers may not be readily available due to scarcity (e.g., high cost or lack of resources to repair broken machines) or unreliable power grids (18). Kangaroo Mother Care/Skin-to-skin care (KMC/STS), in which an adult caregiver holds the newborn bare skin-to-bare skin, thereby transferring warmth to the neonate, preventing hypothermia, is a newborn care initiative that has been targeted for global scale up by a number of international partners. KMC/STS has been shown to be a low-cost and effective solution to prevent neonatal hypothermia, as well to provide numerous other benefits for both the neonate and the primary caregiver such as supporting improved breastfeeding and bonding between caregiver and child (19–22). However, KMC/STS has faced challenges in regards to widescale adoption and scale-up (23–26). For healthcare providers, it can be perceived that KMC/STS interferes with the regular flow of clinical care and monitoring, such as for vital signs. For adult caregivers serving as the KMC/STS partner to the premature/small baby, KMC/STS can be physically demanding, leading to exhaustion and fatigue, and limiting the caregiver from taking a break and caring for themselves (20, 27–29).

Other devices that aim to address neonatal hypothermia have been developed (30–46); however, they are either incompatible with KMC/STS or vital signs monitoring. There remains a need for a low-cost solution for prevention of neonatal hypothermia that is both compatible with KMC/STS and has the capability to be integrated with evidence-based recommendations for regular measurement of key neonatal vital signs to detect common complications of prematurity such as apnea. As a result, we have developed NeoWarm, a sensor-enabled carrier and swaddling device that both allows for KMC/STS and integrates temperature sensing and key vital signs into a single carrier (47–52). Our device will not reduce time spent in KMC/STS, but support and augment KMC/STS. Our previous studies have validated the ability of NeoWarm to warm hypothermic (35°C) tissue phantoms (bottle filled with water) to 37°C (normal human body temperature) and maintain this temperature for 2 h with no overheating (47, 48). These studies have also validated our ability to miniaturize our vital signs monitoring technology into a package that can be integrated into the neonatal carrier (53–58).

Aside from tissue phantoms, which we used in our previous work, current neonatal models for evaluating thermal care focus primarily on thermal manikins which have integrated heating elements and other electronics to mimic heat transfer and flow in the human body. However, while thermal manikins can mimic total heat loss (59, 60), they cannot simulate safety issues such as tissue response and burning. Therefore, a living biological model is necessary to evaluate NeoWarm's ability to safely warm a neonate without causing burns or visible discomfort, which is particularly important as premature neonates have significantly thinner and more fragile skin (61). Currently, there is no standard animal model specifically for neonatal hypothermia. Neonatal piglets are a promising model for neonatal hypothermia as they are of the approximate size and weight of prematurely born/small human neonates (1–2.5 kg), have similar cardiovascular systems, approximate skin composition, and similar core body temperatures (38.6°C–39.7°C in piglets vs. 36.5°C–37.5°C in humans) (13–15, 62, 63). Combined, these characteristics result in humans and piglets having similar thermoregulatory capabilities (64–70). Thus, for the current study, neonatal piglets were selected as a pre-clinical model to test the safety and efficacy of NeoWarm to ameliorate induced hypothermia.

## Materials and methods

This study was approved by the Purdue University Institutional Animal Care and Use Committee (PACUC) under protocol #2008002063. Veterinary technicians monitored the piglets during the entire experiment via visual inspection and palpitation to ensure the piglets’ well-being.

### Inclusion criteria

Piglets were acquired from the Purdue Animal Sciences Research and Education Center (ASREC) farm. Inclusion criteria were that piglets were less than 120 h old, greater than 0.75 kg, less than 2.5 kg, and were within 1°C of a healthy initial core temperature (38.6°C–39.7°C) when they arrived our facility as measured using a commercial rectal thermometer (Part Number: VET-TEMP® DT-10, Advanced Monitors Corporation, San Diego, California, USA). For our study, we utilized convenience sampling, and thus, did not select based on sex. When two trials were able to be run on the same day, we selected litter mates, when possible, to minimize variability between piglets.

### Acquiring piglets from the ASREC farm

Piglets were obtained from their birthing pen at the farm to start each day. Most days, two piglets were available from the same litter, allowing us to run two trials concurrently. Birthweight and initial temperature were acquired for each piglet at the farm. The piglets were then transported 30 min to the testing facility in separate travel crates in the back of an air-conditioned van. Temperature and weight were collected upon arrival at the testing facility.

### Pen setup

**Figure 1:**
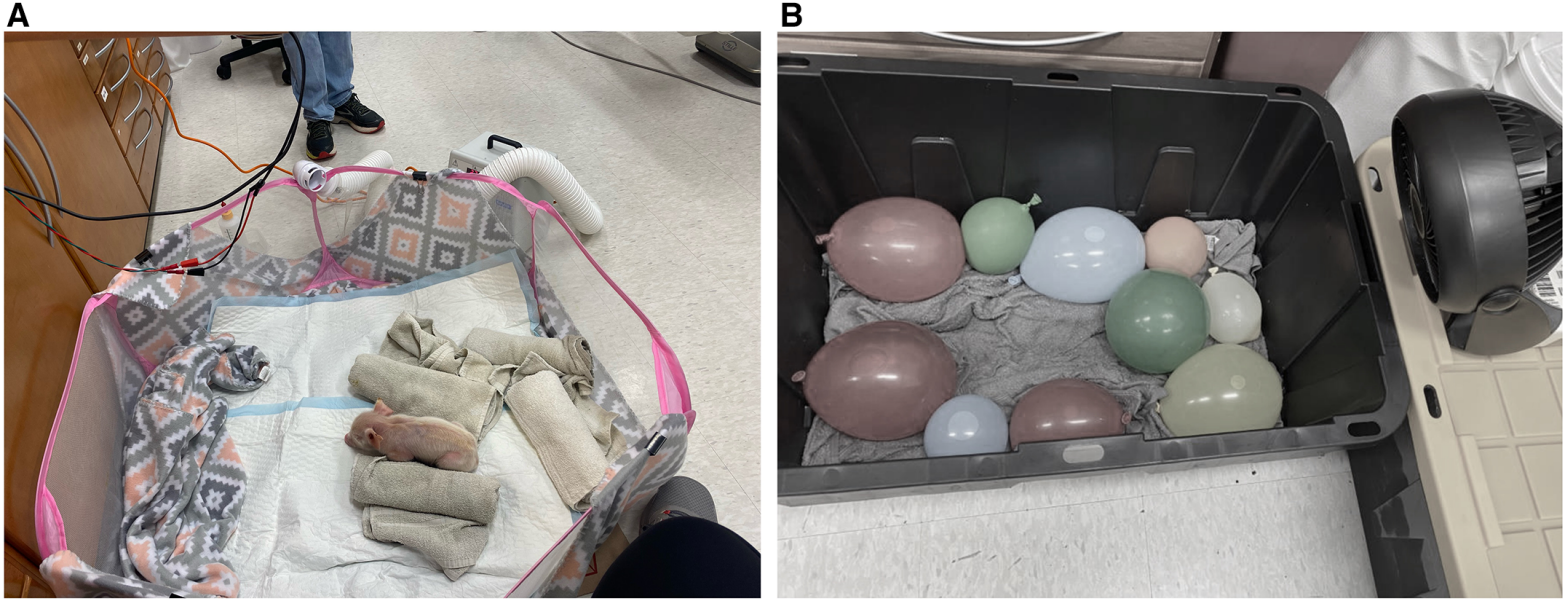
(**A**) Image of neonatal piglet in the pen. (**B**) Image of the cooling box with cold water-soaked towels, cold water-filled balloons and small fan.

As shown in Figure 1A, a roughly 4-ft diameter soft-sided pen was chosen to allow the piglets mobility while keeping them contained. A sheet of cardboard was placed underneath the pen to insulate the bottom of the pen from the cold laboratory floor. Additionally, the inside of the pen was lined with a layer of fleece blankets and absorbent pads to provide more insulation from the cold floor, give the piglets a more comfortable surface to lay on, and to make cleanup easier. Importantly, the piglets were not able to snuggle into the blankets, preventing them from getting additional warmth from their environment. Care was taken so that there were no drafts in the 17.8°C ± 0.6°C room. To prepare for the risk of dangerous levels of hypothermia or deleterious health impacts as determined by the veterinary technicians monitoring the study, we had a heat lamp fixed above the pen (turned off), warm rice pillows, a warm air blower, and additional fleece blankets on standby.

### Temperature phase: cooling

Most days, two piglets were available from the same litter from the farm at a time. Both piglets were placed into a 27-gallon plastic tub lined with ice water-soaked towels and cold water-filled balloons as shown in Figure 1B. Small fans were positioned to blow air into the tub and onto the piglets. The piglets were left in the tub until they reached a core body temperature below 36.5°C or three hours had passed without their temperature dropping below 36.5°C.

### Temperature phase: heating

Piglets were divided into “NeoWarm” or “ambient environment” groups based on three distinct scenarios. (1) The first of the two litter mates to drop below 36.5°C was placed in NeoWarm with the heating pads set to 40°C. The other piglet was placed back into its original pen to warm itself as a control. (2) If both piglets dropped below 36.5°C at the same time or (3) neither piglet dropped below 36.5°C, the piglet with the lower core temperature was placed inside NeoWarm and the other piglet was placed into the 17.8°C ± 0.6°C pen in the ambient environment group.

### Data collection

Piglet core temperature was collected with a rectal thermometer every 15–45 min after their arrival at the testing facility. The veterinary technicians continually monitored the piglets and fed them when their behavior indicated they were hungry. The ambient temperature of the room was set at the beginning of the day, recorded at the beginning of each trial, and verified at least twice throughout the trial, once in the middle and once at the end. The trial was concluded when the piglets’ rectal temperature was stable and did not vary more than 1°C in approximately two and a half hours.

### Euthanasia

Piglets were anesthetized with nitrogen gas, and then Euthasol was used by the veterinary technicians to humanely euthanize the piglets at the end of each trial according to approved animal safety and welfare protocols. Fifteen (15) piglets were euthanized via the intravenous route. One (1) piglet was euthanized via the intracardiac route after two separate veterinary technicians were unable to obtain intravenous access. Prior to euthanasia, a final temperature was taken.

### Data analysis

Once the data points were collected, final temperatures were compared between the NeoWarm group and the ambient environment group. Student's *t*-test was used to compare averages across the two groups. Plots were generated using MATLAB (71) and statistics were computed in MATLAB (R2020b, MathWorks, Natick, Massachusetts, United States) or Microsoft Excel (Microsoft Corporation, Redmond, Washington, United States).

## Results

### Piglets

**Figure 2:**
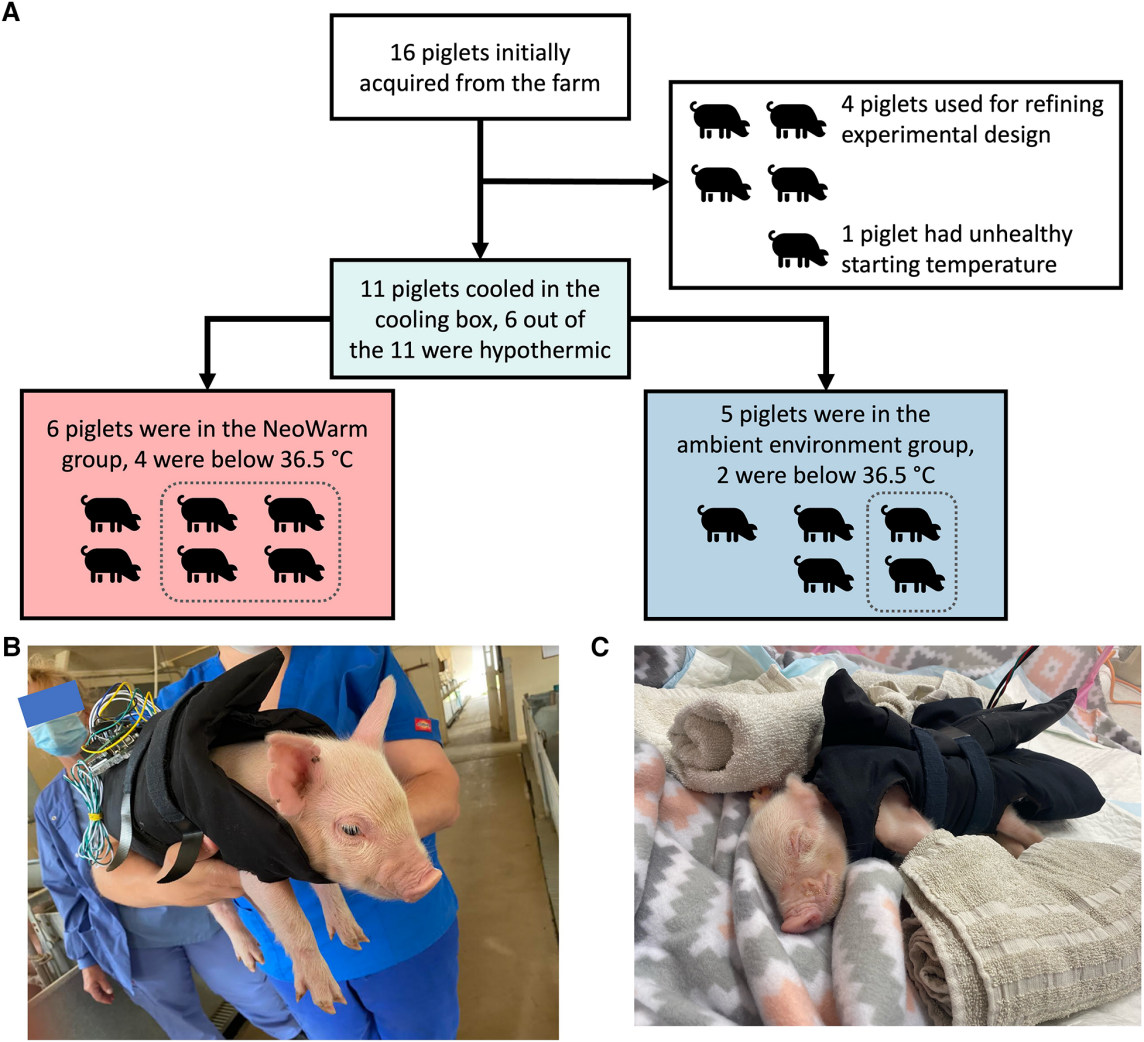
(**A**) Flow chart detailing how pigs were grouped for each stage of the experiment. Images of piglet snuggled in NeoWarm (**B**) at the farm and (**C**) in the pop-up pen.

Sixteen (16) piglets were initially acquired from the ASREC Farm (Figure 2). Four (4) were used to help develop the experimental design and were excluded from final analysis. One (1) additional piglet was removed from analysis as it did not meet the inclusion criteria of having a healthy starting temperature at our facility within 1°C of 38.6°C, with its starting temperature being 32.4°C.

The remaining 11 piglets were enrolled in the study. There were five male piglets and six female piglets in this study. The piglets weighed an average of 1.17 ± 0.16 kg, were all younger than 120 h (five days) old and were within 1°C of a healthy initial core temperature (38.6°C–39.7°C) (62, 63) when they arrived our facility as measured using a commercial rectal thermometer.

### Temperature phase: cooling

The average final temperature at the end of the cooling phase of all the piglets was 36.4°C ± 1.1°C (*n* = 11). Six (6) out of 11 piglets fell below 36.5°C within an average time of 73.2 ± 35.4 min (*n* = 6). Five (5) piglets never dropped below 36.5°C even after three hours in the cold tub. The average final temperature at the end of the cooling phase of the six piglets that did drop below 36.5°C was 35.6°C ± 0.8°C (*n* = 6), while the average final temperature at the end of the cooling phase of the five piglets that did not drop below 36.5°C was 37.2°C ± 0.6°C (*n* = 5). The Student's *t*-test indicates that the difference in final temperature between the six piglets that did drop below 36.5°C and the five piglets that did not drop below 36.5°C is statistically significant (*p* < 0.0051).

Of the six piglets that had final temperatures below 36.5°C at the end of the cooling phase, four of them were placed in NeoWarm, along with two piglets that did not have a final temperature below 36.5°C. The average final temperature of the six piglets placed in NeoWarm at the end of the cooling phase was 36.0°C ± 0.9°C (*n* = 6), while the average final temperature of the five piglets that were not placed in NeoWarm at the end of the cooling phase was 36.8°C ± 1.2°C (*n* = 5). The Student's *t*-test indicates that the difference in final temperature between the six piglets that were placed in NeoWarm and the five piglets that were not placed in NeoWarm at the end of the cooling phase was not statistically significant (*p* = 0.24). We do note that piglet A4 dropped below 36.5°C after the cooling phase. This piglet was not included in the statistics above since it dropped below 36.5°C in the warming phase, not the cooling phase.

All 11 piglets fell below 38.6°C after being in the cooling box.

### Temperature phase: warming (NeoWarm or ambient environment)

**Figure 3:**
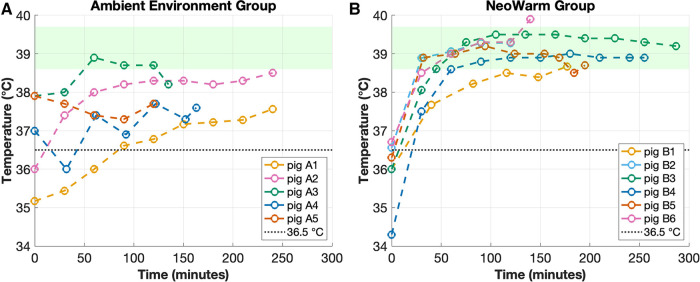
(**A**) Temperature of piglets that were in the ambient environment group vs. (**B**) piglets that were in the NeoWarm group. The dashed line depicts the 36.5°C cooling box threshold and the green shaded region highlights the normothermic temperature range for piglets (38.6°C–39.7°C).

The average final temperature of all 11 piglets at the end of the heating phase was 38.6°C ± 0.8°C (*n* = 11). The average final temperature of the six piglets that were placed in NeoWarm was 39.2°C ± 0.4°C (*n* = 6) after an average time of 183.8 ± 71.2 min (*n* = 6). As shown in Figure 3B, of the six piglets placed in NeoWarm, the highest final temperature of 39.9°C was observed in piglet B6, and the lowest final temperature of 38.7°C was observed in piglet B1. Piglet B6 exceeded the normothermic cutoff of 39.7°C by 0.2°C, having a final temperature of 39.9°C at the end of the heating phase. NeoWarm warmed the piglets up to 38.6°C at a rate of +3.42°C/min. All six piglets that were placed in NeoWarm reached normothermic temperatures at the end of the heating phase, having final temperatures equal to or above 38.6°C (Figure 3B).

The average final temperature of the five piglets in the ambient environment group was 37.9°C ± 0.4°C (*n* = 5) after an average time of 179.6 ± 57.3 min (*n* = 5). As shown in Figure 3A, of the five piglets, the highest final temperature of 38.5°C was observed in piglet A2, and the lowest final temperature of 37.6°C was observed in piglets A1 and A4. None of the five piglets in the ambient environment group had final temperatures in the normothermic range (Figure 3A). Piglet A3 had a peak temperature within the normothermic range, but it was not maintained. Its final temperature dropped below 38.6°C at the conclusion of the trial.

The Student's *t*-test indicates that NeoWarm did warm the piglets to a statistically significant higher core body temperature than the piglets in the ambient environment group (*p* < 0.001). No piglet in either group had any observable burns or skin irritations, as visually and physically assessed by the veterinary technicians.

Critically, all six piglets that were placed in NeoWarm reached normothermic temperatures at the end of the heating phase, having final temperatures equal to or above 38.6°C, while none of the five piglets in the ambient environment group reached normothermia, having final core body temperatures less than 38.6°C (Figure 3). The Pearson's correlation coefficient indicated that there was no significant correlation between weight and final temperature for piglets in the NeoWarm (r = −0.35, *p* = 0.50) or ambient environment groups (r = −0.01, *p* = 0.99) (Table 1).

**Table 1:** Piglet demographic information and temperatures at the end of each temperature phase.

			Cooling		Warming	
	Gender: M/F	Weight: Avg ± Std	Avg ± Std	Min to max	Avg ± std	Min to max
Total *n* = 11	5/6	1.17 ± 0.16	36.4 ± 1.1	34.3–37.9	38.6 ± 0.8	37.6–39.9
NeoWarm *n* = 6	4/2	1.17 ± 0.12	36.0 ± 0.9	34.3–36.7	39.2 ± 0.4	38.7–39.9
Ambient *n* = 5	1/4	1.17 ± 0.21	36.8 ± 1.2	35.2–37.9	37.9 ± 0.4	37.6–38.5
*p*-value (NeoWarm vs. ambient)		0.99	0.24		<0.001	

All weights are in grams (g) and all temperatures are in degrees Celsius (°C).

## Discussion

### Defining normothermia and hypothermia for this study

The reported normal core body temperature (normothermia) for piglets varies, but the most reliable range we found in the literature was 38.6°C–39.7°C (62, 63). We found additional sources that suggest that a temperature range of 38°C–40°C was acceptable, but those metrics were less commonly reported in the literature (72, 73). Therefore, we used the temperature range of 38.6°C–39.7°C as our normothermic range for the piglets in this study. Consequently, below 38.6°C was considered hypothermic for the piglets. For comparison, the normothermic range for newborn humans is 36.5°C–37.5°C with hypothermia defined as core body temperature below 36.5°C (16).

We cooled the piglets below 36.5°C, instead of simply below 38.6°C, to show direct translatability of NeoWarm's performance in this pilot study to the human use-case and to demonstrate NeoWarm's warming capabilities from moderate hypothermia to normal body temperature. We also note that NeoWarm was modified to warm the piglets to their normothermic temperatures of 38.6°C–39.7°C, not the human normothermic temperature of 36.5°C–37.5°C. Human normothermic temperatures would still be severely hypothermic for the piglet and would not allow us to compare NeoWarm's effectiveness to the piglets’ own thermoregulatory capabilities as the piglets would naturally try to warm themselves up to 38.6°C–39.7°C. However, NeoWarm can be readily modified in software to regulate to 36.5°C–37.5°C for the human use-case as we have demonstrated in our previous work using tissue phantoms (48).

### Neowarm's device modifications

We modified NeoWarm from our previously published version (48) in order to accommodate some of the significant differences between piglets and human newborns. An obvious difference affecting form factor is that piglets are four-legged animals while humans walk upright on two legs. Also, critical for this study, piglets are mobile at (or very near) birth. Our prior form factor, resembling more of an infant carrier or baby wrap (48), impeded the piglets’ ability to walk, resulting in distress. Therefore, to allow the piglets to walk and sleep in NeoWarm while remaining thermally insulated, we redesigned the form factor to incorporate holes for the piglets’ legs. We used the same heating pads and microcircuitry that we used in the previous version of NeoWarm. Re-configuring NeoWarm with holes for the piglets’ legs allowed the piglets to walk freely around the pen. To further accommodate their need to walk, we ran NeoWarm from a benchtop power supply, with lightweight cables extending from the power supply to NeoWarm, rather than battery packs, as the weight of the batteries was too heavy for the piglets to move around comfortably. Future work will focus on making NeoWarm more power efficient, so that we can ultimately run the device using lightweight battery packs.

### Modified temperature control algorithm

In our previous iteration of NeoWarm, we monitored the temperature of the heaters as well as the temperature of the tissue phantom using separate temperature sensors (48). Monitoring the temperature of the heaters allowed us to ensure the heaters did not exceed 38°C, the maximum recommended water bath temperature for newborns (16, 74, 75). Monitoring the temperature of the tissue phantom allowed our algorithm to regulate power delivered to the heaters such that the tissue phantom did not exceed the desired setpoint of 37°C, the average human neonatal core body temperature (16).

However, in our present study with piglets, we were unable to continuously monitor the temperature of the piglets using NeoWarm. We utilized intermittent measurements (every 15–45 min) of core body temperature, via a rectal thermometer. Unlike human newborns where we can measure skin temperature at several locations (abdomen, axilla, etc.) to reliably estimate core body temperature (76–80), core body temperature in piglets can only be reliably measured from the rectum (81). Infrared thermometers have been demonstrated to show errors of up to 2°C when estimating core body temperature from the forehead, abdomen, or other locations on the skin. We attempted to continuously monitor both the heater temperature and the piglet's instantaneous rectal temperature in this study; however, the piglets found the temperature sensor very uncomfortable and restrictive even though the sensor was very small (1 mm in diameter). Therefore, we were not able to continuously monitor the piglet's rectal temperature. Consequently, we modified our algorithm to regulate the temperature of the heating pad at 40°C, just slightly above the maximum normothermic temperature of the piglets (38.6°C–39.7°C) (62, 63). We chose a setpoint slightly above 39.7°C to help account for any normally occurring heat loss through the material and to the environment. Our modified algorithm does not incorporate instantaneous temperature measurements from the piglet, since we were unable to continuously measure the piglets’ rectal temperatures using NeoWarm. Nonetheless, regulating the temperature of the warmer, without incorporating feedback from the continuous temperature of the neonate is an acceptable approach that has been demonstrated by others in the literature and in commercially available neonatal warmers (34, 43, 44). This approach is only a necessary modification implemented in this study and will likely not be translated to the human use-case since we can reliably measure the temperature of the newborn from various locations on the skin without causing undue distress to the newborn. However, the results from the current study suggest that our modified approach could be employed in the human use case as well, albeit at a lower set temperature.

### Piglets may have better thermoregulatory capabilities than previously thought

This study suggests several potential considerations in utilizing the neonatal pig as an animal model for human neonatal thermoregulatory capabilities. Based on the literature and the experience of the veterinary technicians, we expected that the piglets would be thermally compromised and rapidly enter hypothermia without external intervention while in the 17.8°C ± 0.6°C laboratory environment (82–84). We observed that this was not the case. Two of the four piglets used in development of the experimental design were left in the ambient environment (not being placed in the cooling box or in NeoWarm). These piglets lost heat at a rate of less than 0.01°C/min. Human premature neonates could lose up to 0.3°C/min in cold environments (85), and this was expected to be the case for the piglets as well without any thermal support. The piglets core temperatures were dropping throughout the experiment (<−0.01°C/min), but much more slowly than human neonates [−0.3°C/min for human neonates (85)]. Also, one of these two piglets kept its internal temperature above 38.6°C, never becoming hypothermic even after six hours in the 17.8°C ± 0.6°C laboratory environment. These preliminary trials led us to use the cooling box to more rapidly cool the piglets below hypothermic temperatures within a reasonable time frame and to demonstrate the warming capabilities of NeoWarm. However, with only two piglets used for these preliminary trials, we cannot make any statistically significant claims regarding piglets’ baseline thermoregulatory capabilities, and we suggest further studies into this matter.

### Neowarm is effective at regulating core body temperature

Our results indicate that NeoWarm is effective as a thermoregulatory solution for hypothermic piglets. Our study has demonstrated that NeoWarm can successfully maintain piglets at a safe core body temperature of 38.6°C–39.7°C, indicating NeoWarm can maintain the piglets’ body temperatures without inducing hyperthermia. Only one piglet, piglet B6, exceeded 39.7°C at any point in the study, having a final temperature of 39.9°C. However, we found reports indicating that a core temperature of up to 40°C (72) or even 41°C (86) could also be observed in piglets. Therefore, we deduce that a 0.2°C overshoot would not be a cause for undue concern. All six piglets that were placed inside NeoWarm were warmed to normothermic temperatures (38.6°C–39.7°C) within 67.3 ± 55.3 min (*n* = 6), while none of the five piglets in the ambient environment group had a final temperature equal to above 38.6°C, and only two of the five even reached the broader definition of normothermia of 38°C–40°C reported by other sources (72, 73, 86). Piglet A3 had a peak temperature within the normothermic range, but it was not maintained. Its final temperature dropped below 38.6°C at the conclusion of the trial, indicating that the piglet was not able to properly maintain its temperature on its own.

### Neowarm is safe

Our study demonstrated that NeoWarm is safe. No piglet demonstrated evidence of burns or skin injury by NeoWarm, as visually assessed by veterinary technicians at the end of each trial, and only one piglet overshot the high end of normothermia (39.7°C) and did so only by 0.2°C. Furthermore, we informally observed that the piglets seemed to be very comfortable inside NeoWarm. Piglets inside NeoWarm slept comfortably, while piglets that were left to warm themselves continued to shiver and roam around the pen seemingly looking for warmth. We did observe that piglets tried to wriggle themselves out of NeoWarm when they were at normothermic temperatures (38.6°C–39.7°C); however, piglets also tried to wriggle themselves away from the veterinary technicians when being held.

### Limitations of the study

Due to convenience sampling, our study was limited to only one breed of piglets, and we were not able to match weight, sex, and litter. Our sample size was also small, having only 11 piglets in the study. It was difficult to acquire a large number of piglets for testing, as piglets are not selectively bred year-round. Given that our access was limited to only a few sows and our testing facility could only manage two piglets per weekday, inclusion criteria provided for enrollment of piglets at ages up to 120 h. However, we aimed to enroll piglets in our study within 24 h whenever possible. Additionally, although trained veterinary technicians reviewed the animals and concluded no damage or harm was done to the animal, skin samples were not taken to experimentally confirm their visual inspection.

### Summary

This pilot study presented thermal monitoring and regulation of 11 piglets to demonstrate safety and preliminary thermoregulatory efficacy in a pre-clinical animal model for a novel biomedical device, NeoWarm. Our studies have shown that NeoWarm is able to warm hypothermic piglets to normothermic temperatures (38.6°C–39.7°C) with no more than 0.2°C overshoot. Not only is NeoWarm effective at warming the piglets, but it is also safe. Our study also suggests that neonatal piglets have more mature thermoregulatory capabilities than as might be expected by review of the literature and by seasoned veterinary professionals. In order to better define neonatal piglets as a standard for neonatal human thermoregulation, further studies would be required to define the limits of neonatal piglet thermoregulatory capabilities.

While safety was effectively demonstrated, form factor modifications that were necessary for this study may alter the thermoregulatory capabilities of the device between neonatal humans and piglets. Studies demonstrating NeoWarm efficacy in human neonates will be completed in future work. Future work will also focus on making NeoWarm more power efficient, removing the need of a benchtop power supply, using small, lightweight battery packs instead.

## Data Availability

The raw data supporting the conclusions of this article will be made available by the authors, without undue reservation.
